# Cleavage of the V-ATPase associated prorenin receptor is mediated by PACE4 and is essential for growth of prostate cancer cells

**DOI:** 10.1371/journal.pone.0288622

**Published:** 2023-07-18

**Authors:** Amro H. Mohammad, Frédéric Couture, Isabelle Gamache, Owen Chen, Wissal El-Assaad, Nelly Abdel-Malak, Anna Kwiatkowska, William Muller, Robert Day, Jose G. Teodoro

**Affiliations:** 1 Goodman Cancer Research Center, McGill University, Montréal, Québec, Canada; 2 Department of Biochemistry, McGill University, Montréal, Québec, Canada; 3 Department of Surgery/Urology, Institut de Pharmacologie de Sherbrooke, Université de Sherbrooke, Sherbrooke, Québec, Canada; 4 PhenoSwitch Bioscience, Sherbrooke, Québec, Canada; Universita degli Studi Gabriele d’Annunzio Chieti e Pescara, ITALY

## Abstract

Phosphatase and tensin homolog (PTEN) mutation is common in prostate cancer during progression to metastatic and castration resistant forms. We previously reported that loss of PTEN function in prostate cancer leads to increased expression and secretion of the Prorenin Receptor (PRR) and its soluble processed form, the soluble Prorenin Receptor (sPRR). PRR is an essential factor required for proper assembly and activity of the vacuolar-ATPase (V-ATPase). The V-ATPase is a rotary proton pump required for the acidification of intracellular vesicles including endosomes and lysosomes. Acidic vesicles are involved in a wide range of cancer related pathways such as receptor mediated endocytosis, autophagy, and cell signalling. Full-length PRR is cleaved at a conserved consensus motif (R-X-X-R↓) by a member of the proprotein convertase family to generate sPRR, and a smaller C-terminal fragment, designated M8.9. It is unclear which convertase processes PRR in prostate cancer cells and how processing affects V-ATPase activity. In the current study we show that PRR is predominantly cleaved by PACE4, a proprotein convertase that has been previously implicated in prostate cancer. We further demonstrate that PTEN controls PRR processing in mouse tissue and controls PACE4 expression in prostate cancer cells. Furthermore, we demonstrate that PACE4 cleavage of PRR is needed for efficient V-ATPase activity and prostate cancer cell growth. Overall, our data highlight the importance of PACE4-mediated PRR processing in normal physiology and prostate cancer tumorigenesis.

## Introduction

Prostate cancer (PCa) is characterized by multiple genetic aberrations that enable cells to grow and evade cellular checkpoints [1]. One of these genetic alterations targets the Phosphatase and tensin homolog (PTEN) [2]. PTEN is a classical tumor suppressor protein with both lipid and protein phosphatase activities [3]. By controlling the phosphatidylinositol 3-kinase (PI3K)/Akt pathway, PTEN regulates many downstream cascades that prevent apoptosis and enhance proliferation in PCa cells [4].

In a previous study, we reported that PTEN controls the expression and secretion of the Prorenin Receptor (PRR) and its processed form, the soluble Prorenin Receptor (sPRR), in PCa cells [5]. The PRR gene (*ATP6AP2*), located on the X chromosome, encodes a 350-amino acid protein with four domains: signal peptide, extracellular, single transmembrane, and cytosolic domains [6]. PRR can be processed by a proprotein convertase (PC) family member producing two fragments: sPRR, and a membrane-associated 8.9-kDa C-terminal fragment (M8.9) [7].

The discovery of PRR has provided a mechanism for how catalytically inactive prorenin is able to cleave angiotensinogen to angiotensin to regulate blood pressure [8]. However, several potential mechanisms have been proposed linking PRR to oncogenesis. The M8.9 fragment of PRR associates with the V-ATPase complex and is required for V-ATPase function [6]. In addition, PRR and the V-ATPase complex were shown to be required for Wnt/β-Catenin signaling [9]. Although PRR is clearly implicated in oncogenesis, the importance of PRR processing by members of the PC family on V-ATPase activity is not well understood.

The PC family of serine proteases include furin, PC1/3, PACE4, PC2, PC4, PC5/6, PC7, PCSK9, and SKI-1/S1P. PCSK9 undergoes autoprocessing and is kept in an inactive form [10]. All these serine proteinases, except SKI-1, can process substrates with an optimal recognition sequence of R-X-K-/R-R↓ or a minimal recognition sequence of R-X-X-R↓ [11]. Although these PCs recognize a similar motif, their intracellular routing, governed by their C-terminal domains, can ensure that different PCs do not cleave the same protein [12], albeit with some redundancy [12,13]. Further, PC expression is tissue-specific and PC members will process different substrates depending on their localized expression. Furthermore, these processes become altered in disease states, such as cancer [14].

Different PCs are overexpressed in different cancer types [15,16]. By activating different substrates, PCs may be involved in tumorigenesis [17–19]. Amongst the PC family members, the importance of furin and PACE4 are the most studied in carcinoma [18]. Furin and PACE4 substrates include membrane-type matrix metalloproteinases (MT-MMPs), which can be utilized by cancer cells for extracellular matrix (ECM) remodeling; adhesion molecules, which can be useful to cancer cells in metastasis; and growth factors and receptors like the IGF-1 receptor, PDGF-A, -B, TGF- β, and VEGF-C, -D, which enhance proliferation [19].

Knockdown of PACE4 in PCa cells shows the strongest inhibition of cell growth when compared to knockdown of other PC members, and significantly inhibited growth of tumor xenografts [16]. Furthermore, inhibition of PACE4 using small molecules was shown to inhibit both the proliferation of PCa cells and tumor growth [20,21]. Together, these findings suggest that PACE4 may have an important role in activating specific substrates that promote PCa cell growth [22]. Although there is considerable evidence that PACE4 is implicated in tumor progression, few substrates have been linked to the activity of PACE4.

We previously reported that PTEN is a regulator of PRR expression and sPRR secretion [5,23]. PTEN loss increases PRR expression and sPRR secretion in PCa cells [5]. We further showed that sPRR levels in urine increase with Gleason grade, indicating that enhanced PRR cleavage in PCa cells may be oncogenic [5]. However, PTEN regulates sPRR secretion by controlling both PRR cleavage and by limiting full-length PRR expression [5]. However, it remains unclear if PC cleavage of PRR is necessary for V-ATPase complex function.

In the current study, we explore the relationship between PTEN, PACE4, and PRR in PCa cells using *in vitro*, mouse models, and human samples. In summary, we demonstrate that PACE4 cleavage of PRR is essential for optimal V-ATPase complex activity in PCa cells.

## Materials and methods

### Cell lines, viruses, and vectors

LNCaP and LNCaP C4-2 cells described in [24], PC3, and DU145 were acquired from Dr. Peter Siegel laboratory at the Goodman Cancer centre at McGill university (Montreal, Canada). The PC knockdown cell lines were described in Couture et al., 2012 [16] and were generated following the procedure detailed in D’Anjou et al., 2014 [25]. HEK-293T cells (ATCC #: CRL-3216) were grown in DMEM media supplemented with 10% FBS and gentamycin. To obtain conditioned media, cells were infected and, 24 hours later, were washed in PBS and incubated in fresh RPMI containing 1% insulin, transferrin, and selenium (ITS, Wisent). Conditioned media was then collected after 24 hours of conditioning. Cell lysates (CL) were obtained by harvesting cells and boiling in 1X Laemmli buffer. Equal amounts of protein from cell lysate and conditioned media were resolved by SDS-PAGE. Adenovirus expressing wild-type PTEN, and β-galactosidase (LacZ) have been previously described [26,27]. The PACE4-overexpressing pcDNA3.1 vector was obtained by gene synthesis (QIAGEN) and its preparation is described in Couture et al., 2017 [22]. The PRR-PCDNA3, sPRR-PCDNA3, and M8.9-PCDNA3 vectors were created in the Teodoro laboratory and are described in supplemental methods. PACE4 mRNA targeting was done using shRNA (CCTGGAAGATTACTACCATTT, TRCN0000075250; Sigma Aldrich), and siRNA (GUAUCUAUUUCUACAUAGU, Sigma Aldrich). Furin mRNA targeting was done using shRNA (CCTGTCCCTCTAAAGCAATAA, TRCN0000075238, Sigma Aldrich).

### Drugs, antibodies, and western blots

Bafilomycin A1 was purchased from Invitrogen. The acetyl-dLLLLRVK-amidinobenzylamide (Amba) (C23) PACE4 inhibitor, described in [21], was acquired from Dr. Robert Day (Sherbrooke, Canada). For treatment in cell culture, compounds were solubilized in DMSO before further dilution in water to 1% DMSO. Cells were treated with the different PACE4 inhibitors diluted in serum-free media for 48 hours before cell lysis or media collection. Development of the PACE4 inhibitor ML and the addition of the PEG8 moiety are described in [28]. Western blots were performed using the following antibodies: PTEN (#9559, Cell Signaling), furin (#43996, Cell Signaling), Actin (A2228, Sigma), PRR (GTX114169, GeneTex), and PACE4 (ab151562, Abcam). PRR (Ab40790, Abcam) and PTEN (6H2.1, Dako) antibodies were used on TMAs and PRR antibody (Ab64957, Abcam) was used for mouse IHC. Where indicated, western blots used *TGX Stain-Fr*ee (Biorad) imaging of total lane protein (TLP) as loading control according to manufactures protocols.

### Quantitative real-time PCR

For reverse transcription, 1 μg of RNA extracted using RNeasy (QIAGEN) was treated with DNase I (Invitrogen), reverse-transcribed using Superscript II reverse transcriptase (Invitrogen), and RNase H-treated (Ambion) before performing quantitative PCR using a Stratagene Mx3005P instrument. Relative expression levels were calculated using β-actin as a reference gene with the formula (1+ amplification efficiency)-(CT). At least three independent experiments (n = 3) were performed. Human PC primers used for RT-qPCR are listed in [16]. Mouse primers used are Actin: FWD: 5’ GATCAAGATCATTGCTCCTCCTGAGC 3’, REV: 5’ GCAGCTCAGTAACATTCCGCCTAG 3’, Furin FWD: 5’ ATCAGGAGCCCACGGACCCC 3’, REV: 5’ CACTGCTGCCACTTCCCCGG 3’, PACE4 FWD: 5’ CGGGTGAAGTCGCTGCCTCG 3’, REV: 5’ CGGTCACATCGCCGTCCAGC 3’, PC5/6 FWD: 5’ AGGGATCCCGCTGTTCGGTCA 3’, REV: 5’ CTCCTTCTGCCCAGGACTCTTCCG 3’, PC7 FWD: 5’ GCTCCGGGGAAGCCGAGAGT 3’, REV: 5’ CAGAGGCAGATGGGCAGGCC 3’, and PRR FWD: 5’ GTAAATGTCTTGATCTGCTGTAA 3’, REV: 5’ TTGCAGAAGAGATTAAAAACAGC 3’.

### PRR peptide cleavage analysis and membrane PACE4 inhibition assay

Synthesis of the PRR spanning peptide was performed manually using a standard solid-phase peptide method on TentaGel S RAM-amide resin. Briefly, Fmoc deprotection and coupling were performed with piperidine 20% in DMF (5 and 10 minutes), Fmoc-protected amino acids (3 equiv), O-(7-azabenzotriazol-1-yl)-N, N, N0,N0-tetramethyluronium hexafluorophosphate (HATU, 3 equiv), 1-hydroxy-6-chloro-benzotriazole (6-Cl-HOBt, 3 equiv), and N,N-diisopropylethylamine (DIPEA, 9 equiv). Completion of the reaction was confirmed using the Kaiser test. The peptide was cleaved from the resin using a cocktail of trifluoroacetic acid (TFA)/H_2_O/triisopropylsilane (TIS) (95:2.5:2.5 v/v/v, 20 mL) for 3 hours at room temperature. The products were precipitated in cold diethyl ether, collected by centrifugation, and dissolved and freeze-dried to a white solid. Crude peptides were purified by preparative high-pressure liquid chromatography (HPLC) (VARIAN ProStar). The fractions containing pure product were pooled and lyophilized. The identity and purity of the peptides (97%, at least) were confirmed by HRMS (TripleTOF 5600, ABSciex) and analytical HPLC (Agilent Technologies 1100 system) equipped with a diode array detector with an Agilent Eclipse XDB C18 column.

For cleavage analysis, a 40 μg sample of PRR peptide was incubated at 37°C with recombinant PACE4 or soluble Furin (16U) in 300 μL of buffer (100 mM HEPES buffer, 1 mM CaCl_2_, 1 mM β-mercaptoethanol, and 1.8 mg/mL BSA, pH 7.5) over a period of 2 hours. An empty buffer solution and a solution containing PRR peptide alone were used as controls. Subsequently, analytical HPLC (Agilent Technologies) was promptly used after the incubation period to analyze the samples. The collected fractions were then analyzed using a SELDI-TOF mass spectrometer (Bio-Rad) to identify the cleavage product. LNCaP and DU145 cells (4 X 10^5^ cells) were treated for 1 hour with 10 μM ML or with membrane-impermeable PEG8-ML diluted in serum-free RPMI media.

### V-ATPase Lysotracker DND-99 assay

To measure the effect of PRR knockdown and PRR processing on V-ATPase complex activity, PRR-knockdown LNCaP C4-2 cells were plated after Puromycin selection along with regular LNCaP C4-2 cells on 6-well plates. Regular LNCaP C4-2 cells were then treated with 50 μM PACE4 inhibitor C23, Vehicle (1% DMSO) for 48 hours prior to assaying, or with 100 nM Bafilomycin for 1 hour immediately before addition of *Lysotracker* (negative control). Cells were then washed once with 1X PBS and incubated with regular 15% FBS RPMI 1640 before taken for imaging. Four to six images were captured per well using a Zeiss microscope with a 20X objective and an AxioCam HR camera. For every fluorescent image captured, a bright-field image was also taken for the same cells to count cell number in the field. Analysis was conducted using *Image J* software. Briefly, cells on the bright-field images were manually counted using the *Image J* cell counter plugin. LysoTracker fluorescence signal, which is selective for acidic vesicles within the cell, was then interpreted as mean gray signal value for each image. Mean gray values were computed by *Image J* and divided by the total number of cells counted from the corresponding bright-field images to find the mean gray value per cell.

### Harvesting mouse serum samples quantification of sPRR concentration

Serum samples from *Pace4*^-/-^ mice were collected by cardiac puncture. An ELISA assay (IBL, International, 27782) designed to detect sPRR in mouse and human blood and urine samples as well as in conditioned media, was used to measure sPRR concentrations in *Pace4*^-/-^ and in *PB-Cre4*-*Pten*^loxP/loxP^ mouse serum samples. Procedures followed are detailed in the manufacture’s protocol.

### Mouse model and tissue processing

The *Pace4*^-/-^ mouse model was generated as described in [29]. The design and creation of the *Pten*^loxP/loxP^ mouse model is described in [30]. The resulting *Pten*^loxP/loxP^ mouse strain was then crossed with *Probasin-Cre* (*PB-Cre4*) mice to create the *PB-Cre4*-*Pten*^loxP/loxP^ mouse model [31] used in this study. Generation of *14-3-3σ*^loxP/loxP^ is described in [32]. *14-3-3σ* ^loxP/loxP^ were also subsequently crossed with the *PB-Cre4* to knockout the expression of 14-3-3σ in the prostate tissue. Both the *Pten*^loxP/loxP^ and the *14-3-3σ* ^loxP/loxP^ mouse models were created in a uniform FVB/N genetic background strain. Derived mouse prostate tumors and organ tissue were processed for RNA and cell lysis using the ALLPrep DNA/RNA/Protein Mini Kit (50) (*Qiagen*, 80004).

### LNCaP xenograft

Trypsin-harvested LNCaP cells were mixed with cold Matrigel (BD Biosciences, Bedford, MA) and injected subcutaneously to male Nu/Nu mice (Charles River Laboratories, St-Constant, Canada). Mice were treated daily with 2 or 4 mg/kg of C23 or saline by intraperitoneal injection. After 28 days, mice were sacrificed, and tumor lysates were obtained by grinding flash frozen tissues in liquid nitrogen and solubilized in RIPA buffer with 1x protease inhibitor for 30 minutes. Samples were clarified by centrifugation at 13,000 x g for 10 minutes, supernatants were used for protein quantitation using BCA protein assay (Thermo) and used for Western blot analysis. All mouse procedures were performed in compliance with the McGill facility animal care committee and the Canadian council on animal care guidelines.

### IHC of mouse prostate tissue

At least three 4-micrometer sections were taken from different mouse prostates for each of the genotypes surveyed. Sections were used for H&E staining and for PRR and PACE IHC. Slides were heated for 20 minutes at 60˚C, deparaffinized in xylenes and rehydrated in ethanol gradient. Antigen retrieval was performed using EDTA pH 8.0 (PRR) buffer and sodium citrate pH 6.0 (PACE4). PACE4 slides were further autoclaved in 10 mM citrate buffer pH 6 for 45 min (16 psi, 121 ˚C). To deactivate endogenous peroxidase, 3% H_2_O_2_ was used, and slides were blocked using a protein blocking reagent (Dako) for PRR and an IHC blocking buffer (Pierce) for PACE4, at room temperature. Tissue sections were incubated with primary antibodies diluted in 5% BSA in TBST overnight at 4˚C (Abcam, ab64957, 1:600; PACE4: Abcam, ab151562, 1:400) and then with biotin-conjugated secondary antibodies (Santa-Cruz) for PRR and a secondary HRP-conjugated anti-Rabbit antibody (Biorad) for PACE4. Staining was developed using DAB (Sigma-Aldrich) containing 0.015% H2O2. Counterstaining was performed with hematoxylin.

### Tissue microarrays (TMAs) staining and analysis and the cancer genome atlas

Tissue microarrays (TMAs) were produced from clinical formalin-fixed paraffin-embedded (FFPE) blocks taken from archived prostate specimens from patients who underwent radical prostatectomy between 2006 and 2011 at the Centre Hospitalier Universitaire de Sherbrooke. Clinical information for each patient was obtained by examination of medical records. Four-micrometer sections were produced from TMA blocks. For PRR IHC, TMAs were pre-treated with *DIVA* (DV2004) for antigen retrieval at 125°C for 10 minutes. The TMAs were then blocked for 15 minutes using Enzyme block (S2003) and protein block serum-free (X0909) purchased from DAKO. TMAs were then incubated with PRR antibody (Abcam, Ab40790, 1:1000) for 1 hour and then with 1:500 goat anti-rabbit secondary antibody (111-035-144, Jackson Laboratory). The Chromagen used was DAB (K3468) from *DAKO* and counterstaining was performed using Hematox (3801698) purchased from *Leica*. For PACE4 and PTEN IHC, tissue slices were deparaffinized and stained using a streptavidin-biotin based automated stainer (Dako). Endogenous Peroxidase was quenched using a peroxidase suppressor from a Peroxidase detection kit (Pierce). PACE4 (Abcam, ab151562, 1:400) and PTEN (Dako, 6H2. 1, 1:400) antibodies were diluted in 5% BSA TBST. Slices were then washed and incubated with a secondary anti-Rabbit HRP-conjugated antibody (Biorad, 1:500). After imaging, TMAs were scored for PRR, PACE4, and PTEN staining (0-no staining, 1-weak staining, 2-moderate staining, and 3-strong staining) by 3 independent observers. Scores were then averaged for a qualitative measure for protein expression. Analysis of *ATP6PA2* (PRR), *PCSK6* (PACE4), and *PTEN* expression in human prostate normal and tumor tissues was done by extracting data from TCGA database using the GEPIA2 interface (http://gepia2.cancer-pku.cn/). Log 2 (TPM+1) scale was used to compare expression of the respective genes between normal and tumor tissues.

### Statistical analysis

For all statistical tests conducted in this study, the two-tailed Student t-test was used to test significance. P-values below 0.05 were considered statistically significant.

### Ethics committee approval

To approve the use of patient specimen for biomarker evaluation, all patients from Centre de recherche du Centre Hospitalier de l’Universite de Montreal signed the informed consent form of the ethics committee (CE 15.366) and of the Institutional Review Committee for the use of human resected material at the Centre Hospitalier Universitaire de Sherbrooke (approval #10–017 and #12–151). This study was carried out in strict accordance with the recommendations in the Animal Research Ethics Committee of the University of Sherbrooke. The protocol for the proposed animal experiments and procedures in this study were approved by the committee (approval # 016–08). Mice were housed in ventilated sterilized cages with autoclaved bedding, rodent chow, and water. Mouse procedures were conducted in sterile laminar flow biological cabinets. Animals were routinely examined by animal care techinicians and were sacrificied if they sustained 20% body weight loss or had a tumor larger that 2000 mm in diameter. Euthanasia by cervical dislocation was conducted after anesthesia with ketamine:xylazine intramuscular injection (90:10 mg/Kg) to minimize animal suffering. Animal anesthesia for xenografting was done using isoflurane nebulization. Post procedural signs of pain, dehydration, and distress were routinely monitored and communicated to the veterinarian.

## Results

### PACE4 cleaves PRR in prostate cancer cells

**Fig 1:**
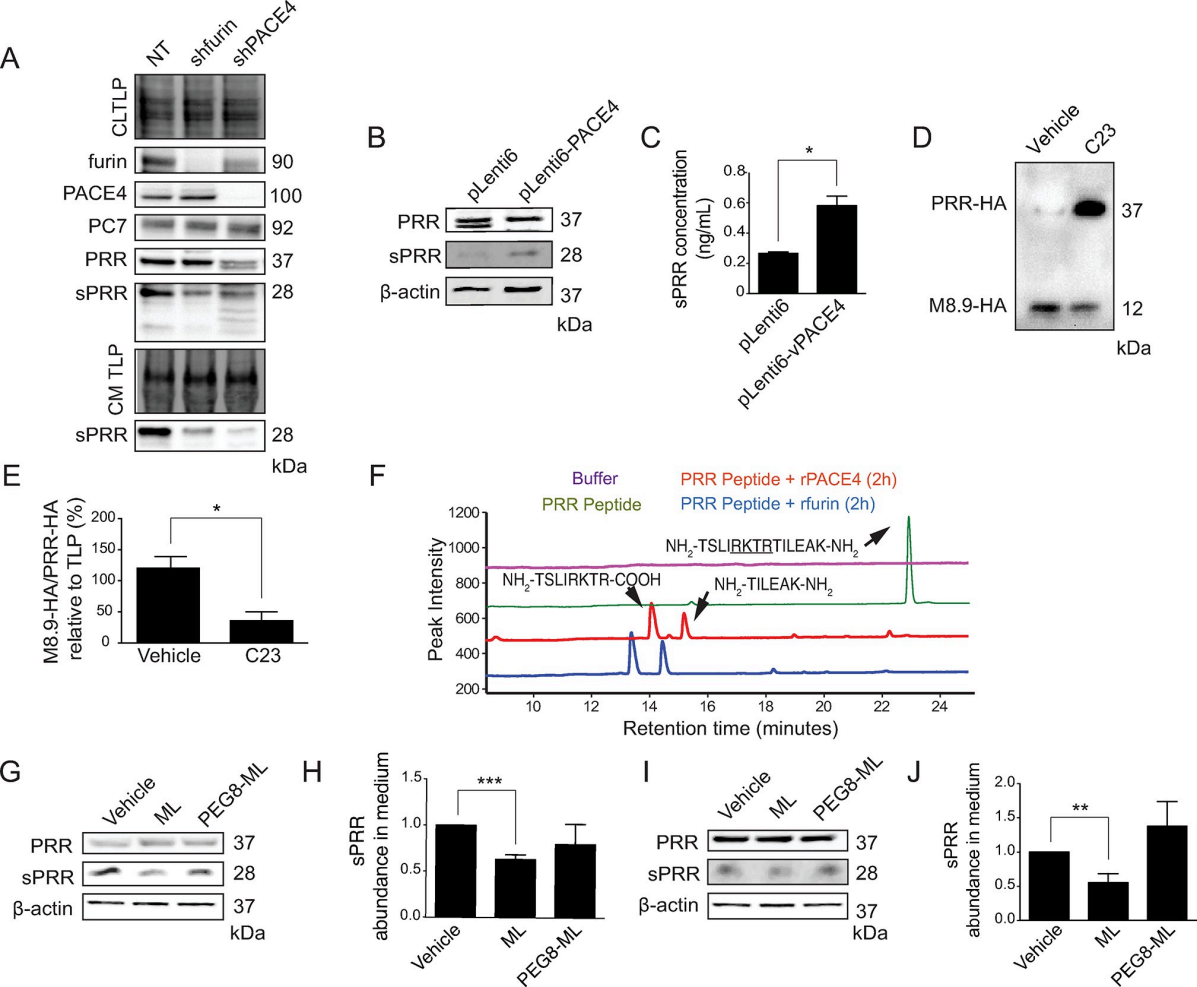
PACE4 cleaves PRR intracellularly in prostate cancer cells. (**A**) Western blot analysis showing Prorenin Receptor (PRR), furin, and PACE4 expression, sPRR secretion, and cell lysate and conditioned media total lane protein (CLTLP, CMTLP) in LNCaP cells infected with a control non-target shRNA (NT), Furin shRNA (shfurin), or PACE4 shRNA (shPACE4). (**B**) Western Blot analysis demonstrating the expression of PRR and secretion of sPRR in LNCaP cells infected with an empty pLenti6 vector or with pLenti6-PACE4 to overexpress PACE4. (**C**) Quantification of sPRR levels in pLenti6 and pLenti-PACE4-infected LNCaP cells (*P<0.05, n = 3). (**D**) Western blot showing the reduction in PRR processing resembled as a ratio of HA-tagged M8.9 (M8.9-HA) to HA-tagged full-length PRR (PRR-HA) in cellular extract of LNCaP cells after a 50 μM PACE4 inhibitor [33] LLLRVK-amidinobenzylamide (Amba) (C23) treatment. (**E**) Corresponding quantification of the ratio of M8.9-HA to PRR-HA standardized over total lane protein (TLP) (*P<0.05, n = 3). (**F**) Analysis of PRR peptide cleavage by recombinant PACE4 (rPACE4) or recombinant furin (rfurin) monitored after a 2-hour incubation by high pressure liquid chromatography (HPLC). Mass spectrometry was done to confirm identity of peptide after cleavage. Cleavage site is underlined on the peptide sequence. Western blot analysis of PRR expression and sPRR secretion and quantification of sPRR secretion after DMSO (Vehicle), 50 μM multi-Leucine peptide (ML) PACE4 inhibitor, or 50 μM PEGylated cell-impermeable ML (PEG8-ML) treatment of DU145 (***P<0.001, n = 3) (**G**, **H**) or LNCaP (**I**, **J**) (**P<0.01, n = 3) cells, respectively. Beta-Actin (β-actin) and TLP were used as loading controls. Data are presented as the mean ± SEM. Statistical tests were conducted using Student’s t test.

PACE4 was previously shown to be overexpressed in PCa cells and its activity was required for tumor growth and progression [16,17,21,22]. Since PRR is a known PC substrate and is required for PCa cell proliferation [5], we hypothesized that it may also be a substrate of PACE4. In Fig 1 A, we show that shRNA-mediated knockdown of PACE4 in LNCaP cells resulted in less processing of PRR and lower levels of sPRR in the conditioned media (CM). Conversely, PACE4 overexpression using lentiviral transduction in LNCaP cells led to increased PRR processing as measured by elevated secretion of sPRR in the CM (Fig 1B and 1C). Previous studies identified a peptide analog, designated C23, as a potent and selective inhibitor of PACE4 [28]. Fig 1D and 1E show that treatment of LNCaP cells transfected with PRR-HA construct with the HA tag on C-terminus with C23 dramatically reduced processing of PRR, as represented by the decreased isolation of the M8.9-HA fragment on gel. To further validate the cleavage of PRR by PACE4, a PRR cleavage site-spanning peptide was incubated *in vitro* with recombinant human PACE4 and soluble furin. Resulting peptides were analyzed using high pressure liquid chromatography (HPLC) followed by mass spectrometry and showed that both PACE4 and furin were able to cleave at the RKTR↓ site (Fig 1F). All PCs have signal peptides and can potentially process substrates intra- and extracellularly [17]. To investigate whether PACE4-mediated cleavage of PRR takes place intracellularly or at the cell surface, cell permeable and impermeable PACE4 inhibitors were used to treat LNCaP cells. Treatment with cell-permeable multi-leucine peptide (ML) PACE4 inhibitor was more effective at inhibiting PRR processing than a cell-impermeable PEGylated version (PEG8-ML), suggesting that PACE4 cleavage of PRR occurs prominently within the cell (Fig 1G–1J). These data suggest that PACE4 is the main PC responsible for intracellular cleavage of PRR in PCa cells.

### PACE4-knockout mice exhibit less processing of PRR

**Fig 2:**
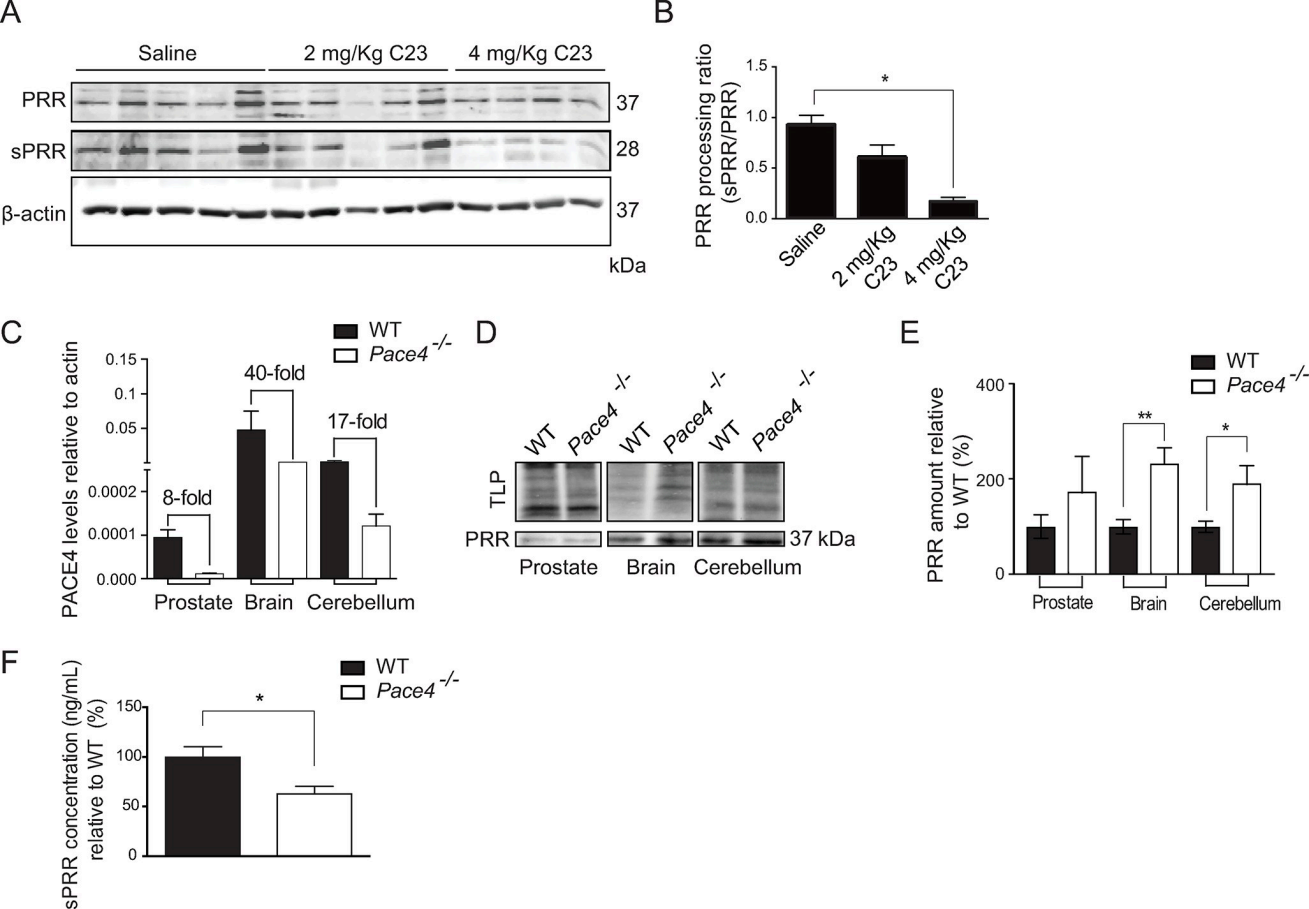
Full-length PRR expression increases and sPRR secretion decreases in the absence of PACE4 in mouse. (**A**) Western blot analysis of prorenin receptor (PRR) and soluble prorenin receptor (sPRR) in prostate tissue extracted from wild type mice treated with saline, 2mg/Kg of PACE4 inhibitor [33] LLLRVK-amidinobenzylamide (Amba) (C23), and 4 mg/Kg C23. (**B**) Quantification of PRR processing in prostate tissue showing less PRR processing in prostate tissue extracted from mice treated with 2 mg/Kg and 4mg/Kg C23 (*P<0.05, n = 4), compared to saline. (**C**) Quantitative PCR measurement of PACE4 expression in prostate, brain, and cerebellum tissue demonstrating the difference in PACE4 expression between wild type (WT) and *Pace4*-null (*Pace4*^-/-^) mice. (**D**) Western blot analysis of prostate, brain, and cerebellum tissue to analyze PRR expression in WT and *Pace4*^-/-^ mice. (**E**) Corresponding quantification of PRR expression in the same tissues (*P<0.05, **P<0.01, n = 8). (**F**) ELISA quantification of plasma sPRR levels in WT (n = 4) and *Pace4*^-/-^ (*P < 0.05, n = 8) mice. Beta-Actin (β-actin) and total lane protein were used as loading controls. Data are presented as the mean ±SEM. Statistical tests were conducted using Student’s t test.

Our previous studies have shown that C23 was able to significantly inhibit the growth of LNCaP xenografts [22]. To examine PACE4 cleavage of PRR in a more physiological system, we analyzed PRR processing in tumour lysates of LNCaP-xenografted mice treated with 2 mg/kg or 4 mg/kg C23 (Fig 2A and 2B). Fig 2B shows that treatment with C23 resulted in a dose-dependent inhibition of PRR processing of PRR within the tumors. PACE4 knockout mice have been derived in previous studies and were shown to be mostly viable although there are craniofacial abnormalities with incomplete penetrance [29]. To further examine the role of PACE4 in PRR processing, we measured expression and processing of PRR in *Pace4*-knockout mouse tissues and plasma (Fig 2C–2F). In a full body *Pace4*-knockout mouse, full-length PRR protein amount was elevated in prostate, whole brain, and cerebellum tissues compared to wild type suggesting that processing was impaired (Fig 2D and 2E). Furthermore, ELISA analysis was used to measure sPRR in the plasma of *Pace4*-knockout mice and showed that sPRR levels were reduced by approximately 40% relative to wild-type animals (Fig 2F). Taken together, these data indicate that PACE4 plays a major physiological role in the *in vivo* processing of PRR.

### PTEN controls PACE4-mediated PRR processing in a mouse model of prostate cancer

**Fig 3:**
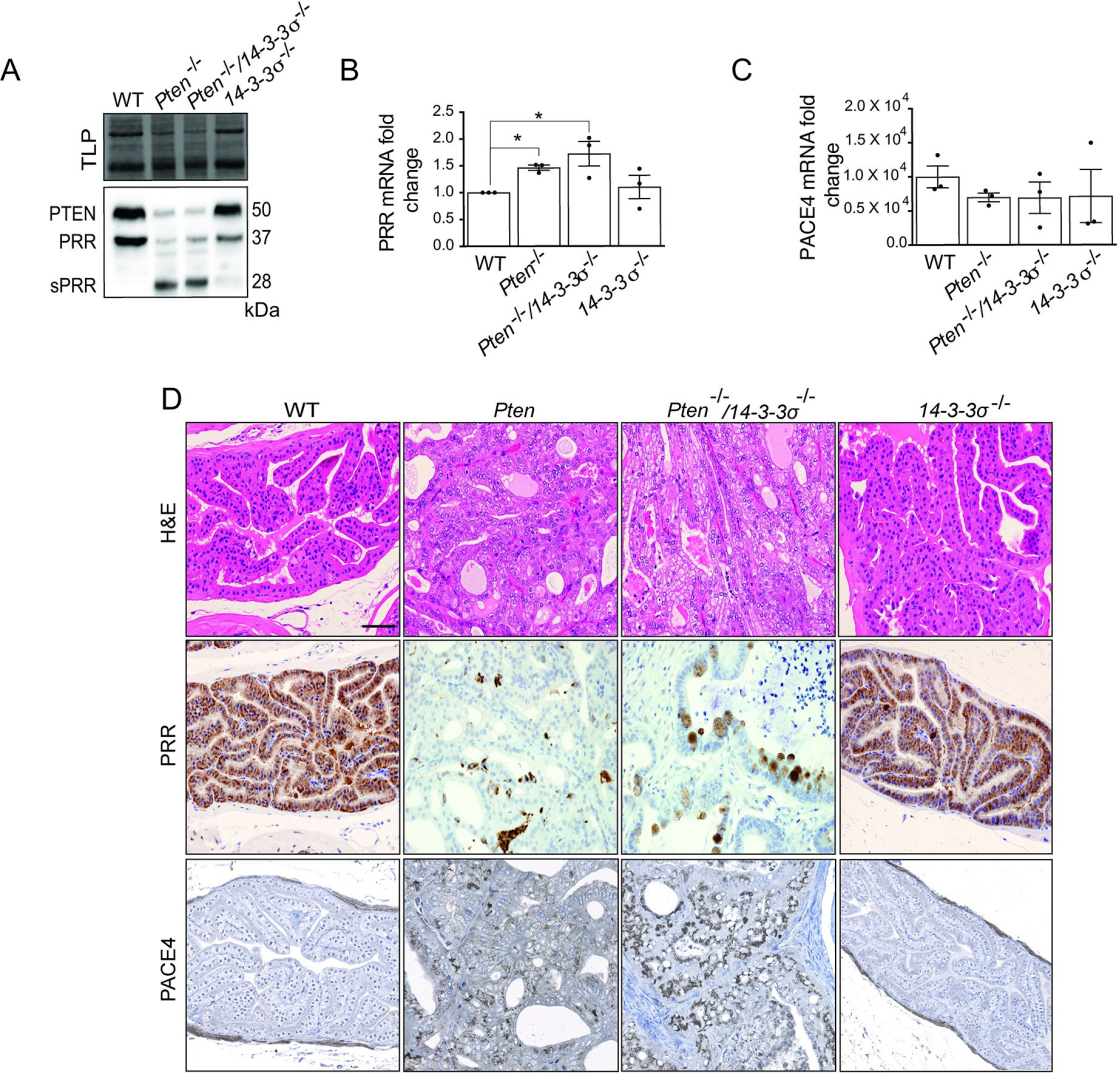
PTEN controls PRR processing and PACE4 protein expression in mouse. **(A)** Western blot analysis of PTEN, PRR, and sPRR in homogenized WT, *Pten*^-/-^, *Pten*^-/-^/*14-3-3σ*^-/-^, and *14-3-3σ*^-/-^ mouse prostate tissue. Total lane protein (TLP) was used as a loading control. Dot plots of (**B**) Prorenin Receptor (PRR) and (**C**) PACE4 mRNA expression levels in wild type (WT), *Pten*^-/-^, *Pten*^-/-^/*14-3-3σ* ^-/-^, and *14-3-3σ*^-/-^ prostate tissue (*P < 0.05, n = 3). Data are presented as the mean ±SEM. Statistical tests conducted using Student’s t test. (**D**) Corresponding representative IHC images of WT, *Pten*^-/-^, *Pten*^-/-^/*14-3-3σ*^-/-^, and *14-3-3σ*^-/-^ mouse prostate sections after H&E, PRR, and PACE4 staining. Scale bar measures 70 μm.

We previously reported that PTEN can control sPRR secretion and PRR expression through regulation of mRNA translation in PCa cells [5]. Therefore, it is possible that PTEN may regulate PRR processing through controlling PACE4 mRNA translation and decrease PACE4 protein levels. To test this possibility, levels of PACE4 and PRR were studied in prostate tissue obtained from 5-month-old wild type, Probasin-Cre/*Pten*^loxP/loxP^, Probasin-Cre/*Pten*^loxP/loxP^/*14-3-3σ*^loxP/loxP^, and *14-3-3σ*^loxP/loxP^ mice. Cre mice/*Pten*^loxP/loxP^ mice have prostate specific deletion of PTEN that results in prostatic intraepithelial neoplasia (PIN) with complete penetrance by 12–20 weeks that ultimately progresses to invasive adenocarcinoma [34]. We examined knockout of a second tumor suppressor gene, *14-3-3σ*^-/-^, to ensure observed effects were specific to PTEN deletion. Immunoblot analysis of prostate tissue lysates shows that deletion of PTEN, but not 14-3-3σ, resulted in a dramatic increase in PRR processing as evidenced by the reduction in full-length PRR and elevation of sPRR (Fig 3A). PRR mRNA levels were significantly increased when PTEN was deleted, whereas PACE4 levels were unchanged in the different conditions (Fig 3B and 3C). H&E staining of sections derived from the same mice displayed disruption of prostatic gland morphology characteristic of metastatic PCa in sections lacking epithelial PTEN expression, whereas sections from wild type and *14-3-3σ*^-/-^ had normal morphology (Fig 3D). IHC staining for PRR in *Pten*^-/-^ prostate sections displayed dramatically diminished PRR immunoreactivity but demonstrated a scattered punctate staining which could be the result of elevated PRR processing (Fig 3D). IHC for PACE4 on the same sections showed increased PACE4 staining within the stroma of PTEN^-/-^ tumors compared to wild type, and *14-3-3σ*^-/-^ sections. These data suggest that loss of PTEN leads to increased PACE4 levels, possibly through a post-transcriptional mechanism.

### PTEN regulates PRR and PACE4 levels in human prostate cancer cells

**Fig 4:**
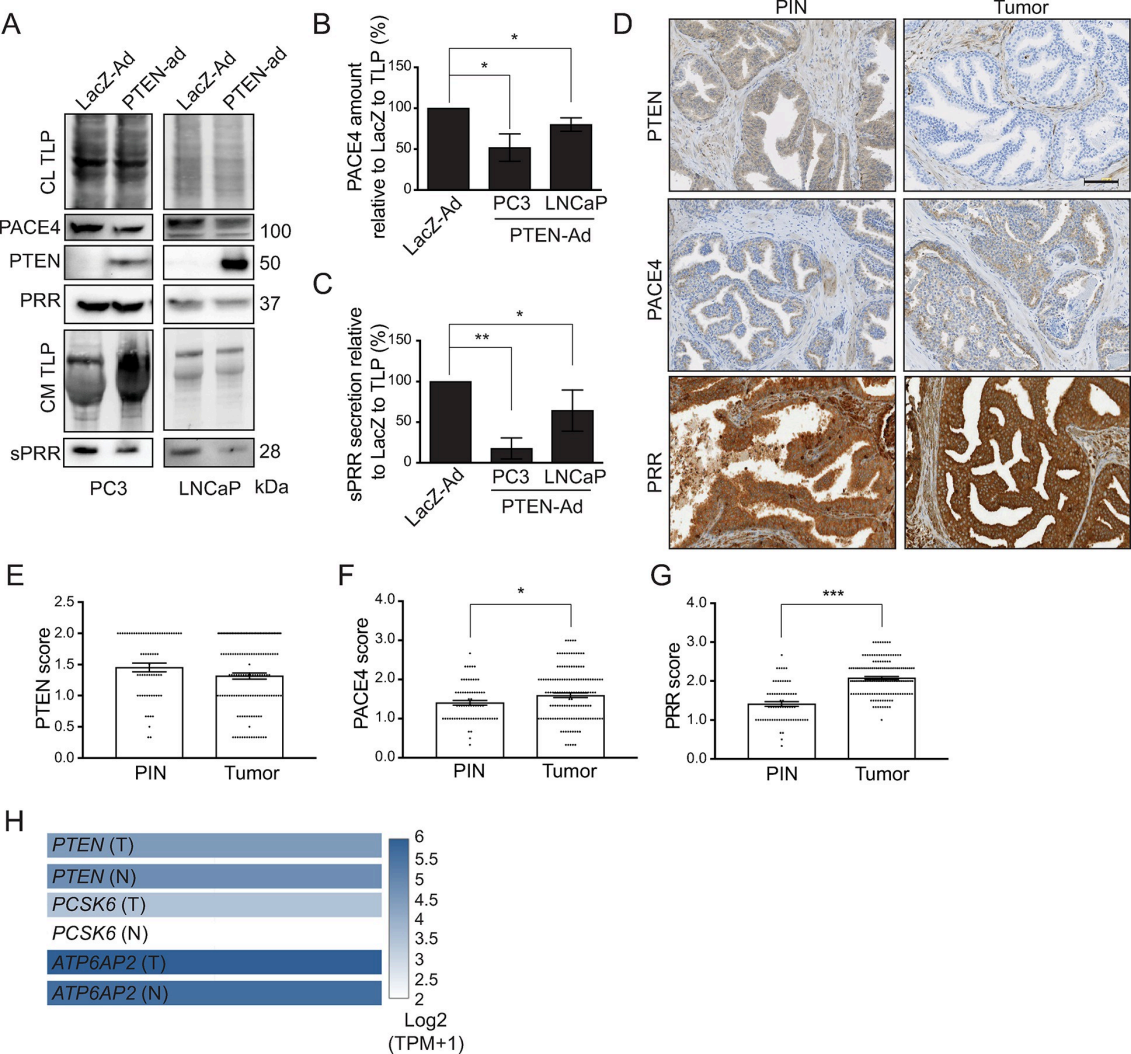
PTEN controls PACE4 expression and sPRR secretion in human prostate cancer cells, and both PACE4 and PRR expressions increase in human prostate tumor tissue. (**A**) Western blot analysis showing less PACE4 endogenous protein expression after adenoviral Phosphatase and tensin homolog (PTEN) infection (PTEN-Ad) in PC3 and LNCaP cells. The same cells also exhibited less Prorenin Receptor (PRR) processing and less soluble Prorenin Receptor (sPRR) secretion in conditioned media (CM). Total lane protein (TLP) was used as a loading control. Quantification of (**B**) PACE4 expression and (**C**) sPRR secretion relative to the corresponding TLP in PC3 and LNCaP prostate cancer cells (*P < 0.05, **P ≤ 0.01, n = 3). (**D**) Representative IHC tissue microarray images of PTEN, PACE4, and PRR staining on prostatic intraepithelial neoplasia (PIN) tissue and tumor tissue. Scale bar measures 100 μm. Dot plots representing the mean of the qualitative score assigned to (**E**) PTEN, (**F**) PACE4, and (**G**) PRR staining on PIN (n = 68) and tumor (n = 105) patient tissue microarrays (*P< 0.05, ***P ≤ 0.001). Data are presented as the mean ± SEM. Statistical tests were conducted using Student’s t test. (**H**) The Cancer Genome Atlas (TCGA) mRNA expression data in Log2 scale of *PTEN*, *PCSK6* (PACE4), and *ATP6AP2* (PRR) in tumor (T) and normal (N) prostate tissue.

To validate the role of PTEN in regulating PACE4 and PRR processing, PTEN was reconstituted in *PTEN*-null PC3 and LNCaP PCa cells using an adenoviral vector. Re-introduction of PTEN in these cells resulted in a decrease in PACE4 protein levels and reduced secretion of sPRR as compared to LacZ expressing control virus (Fig 4A–4C). To provide clinical relevance, tumor microarrays (TMA) containing pairs of matched PIN (n = 68) and tumor (n = 105) sections were stained for PTEN, PRR, and PACE4. Fig 4D and 4E show an insignificant decrease in PTEN amount but an increase in the staining of both PACE4 and PRR in tumor tissue compared to PIN areas (Fig 4D–4G). Furthermore, analysis of gene expression data from The Cancer Genome Atlas (TCGA) comparing PTEN and PACE4 and PRR mRNA expression show a very similar expression pattern as our TMA analysis when comparing normal and tumor prostate tissue (Fig 4H). These observations show that PACE4 and PRR are upregulated in PCa and suggest that these proteins may play a role in the transition from PIN to prostate adenocarcinoma.

### PRR processing by PACE4 is essential for optimal V-ATPase activity in prostate cancer cells

**Fig 5:**
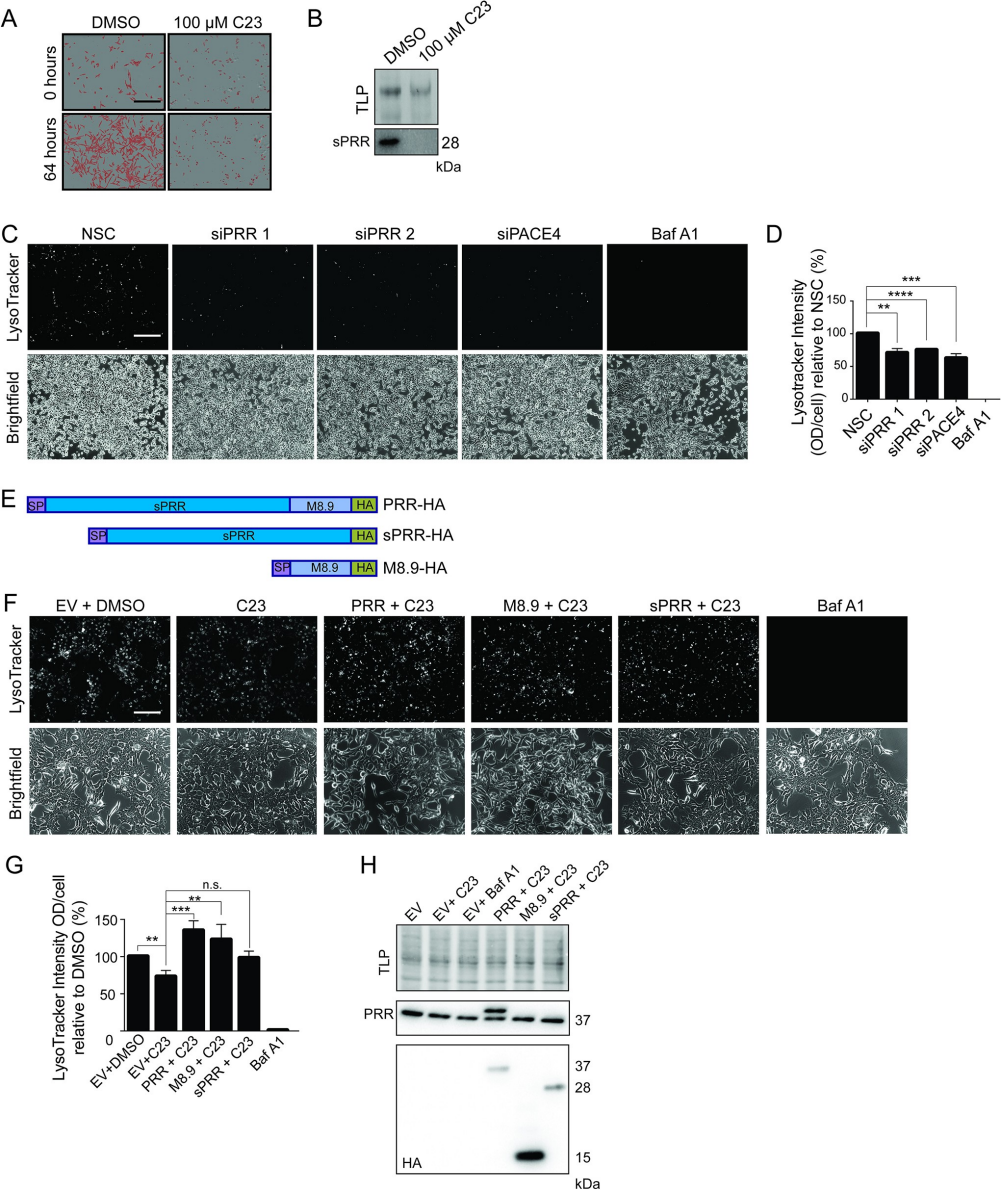
PACE4 inhibition, like PRR knockdown, reduces V-ATPase activity. (**A**) Representative proliferation images of LNCaP exposed to both DMSO or a 100 μM of PACE4 inhibitor [33] LLLRVK-amidinobenzylamide (Amba) (C23) over 0 and 64 hours. (**B**) Western blot analysis of soluble prorenin receptor (sPRR) secretion in conditioned media of LNCaP exposed to DMSO or a 100 μM C23. (**C**) Representative LysoTracker images of LNCaP cells transfected with non-silencing control siRNA (NSC), PRR siRNA 1 and 2, PACE siRNA, or treated with 100 nM Bafilomycin A1. (**D**) Quantification of LysoTracker signal intensity relative to number of cells in brightfield images treated with 1% DMSO (Vehicle), 50 μM C23, 100 nM Bafilomycin A1 (BafA1), or transfected with PRR siPRR 1 or siPRR 2. (**E**) Schematic showing the PRR-HA, sPRR-HA, and M8.9-HA vectors used in panels F, G and H. (**F**) Representative LysoTracker images of LNCaP cells transfected with empty vector (EV) and treated with DMSO, treated with 100 μM C23, transfected with PRR-HA and treated with 100 μM C23, transfected with M8.9-HA and treated with 100 μM C23, transfected with sPRR-HA and treated with 100 μM C23, or treated with 100 nM BafA1. (**G**) Corresponding quantification of LysoTracker intensity relative to cell number in the same treatments (**P ≤ 0.01, ***P ≤ 0.001, **** P < 0.0001, n = 4). (**H**) Western blot analysis of PRR and HA expression in the same treatments. Total lane protein (TLP) was used as loading control. Data are presented as the mean ±SEM. Statistical tests were conducted using Student’s t test. Scale bar measures 100.

PRR plays a vital intracellular role by serving as an accessory factor required for assembly and activity of the V-ATPase complex, which is required for the acidification of intracellular vesicles [35]. As shown in Fig 1D, full-length PRR is very efficiently processed into sPRR and M8.9 in a PACE4-dependent manner in PCa cells, however, the significance of this processing for V-ATPase activity is not known. Fig 5A shows that treatment of LNCaP cells with C23 reduced cell proliferation. Immunoblot analysis of PRR in C23-treated cells showed that drug treatment prevented PRR processing (Fig 5B). To determine the effect of PRR processing on V-ATPase activity, intensity of intracellular acidification was measured using LysoTracker Red probe. Fig 5C and 5D show that siRNA-mediated knockdown of either PRR or PACE4 were similarly able to inhibit V-ATPase activity. As expected, treatment with the V-ATPase inhibitor, Bafilomycin A1 (Baf A1), completely prevented LysoTracker Red staining (Fig 5C and 5D). We then sought to determine the effect of the different constructs of PRR (Fig 5E) on V-ATPase activity in the context of PACE4 inhibition. Treatment with C23 alone significantly inhibited V-ATPase activity suggesting that PRR processing is required for optimal activity (Fig 5F). Since inhibition of PRR processing was able to inhibit V-ATPase activity, we then determined if overexpression of PRR or its processed forms could reverse C23-mediated inhibition. Constructs expressing full-length PRR-HA, sPRR-HA, or M8.9-HA were transfected into C23 treated cells and stained with LysoTracker Red (Fig 5E-5H). Transfection with PRR-HA and M8.9-HA but not sPRR-HA were able to rescue lysotracker signal in the presence of C23 (Fig 5F and 5G). Expression of each of the constructs was confirmed by anti-HA immunoblot (Fig 5H). Taken together, these data show that PACE4-mediated processing of PRR is required for efficient V-ATPase activity and may be an important mechanism of action for PACE4 inhibitors.

## Discussion

In this study, we addressed the significance of PRR processing on the biology of PCa cells. Importantly, we show for the first time that PACE4 is the major PC that cleaves PRR in PCa cells. We observed that PACE4 knockout mice have about 40% less circulating sPRR indicating that PACE4 is a major PC that processes PRR. PACE4 is likely not the only PC that cleaves PRR and other studies have shown that furin [36], SKI-1/S1P [37], and ADAM19 [38] can also process PRR suggesting that PRR processing is likely cell and/or tissue specific. Cancer cells may favour certain PCs over others to cleave substrates or may alter intracellular localization of substrates or proteases [39].

Our previous studies have shown that sPRR secretion is regulated downstream of the PTEN tumor suppressor and is upregulated in both PCa and breast cancer [5,23]. Our current study shows that this effect may in part be due to elevated PACE4 amount following loss of PTEN. In mouse prostate with abrogated PTEN expression, there is a dramatic reorganization of PRR staining that may be due to processing of full-length PRR and shedding of sPRR into the tumor microenvironment and circulation (Fig 3). Analysis of TMA data as well as TCGA gene expression analysis both showed that PACE4 expression levels are elevated in PCa (Fig 4). The involvement of PACE4 in PCa progression has been previously reported [22]. PACE4 was shown to have an alternative spliced form (PACE4-altCT) that was predominant in several cancer types including PCa and displayed enhanced autoactivation [22]. Inhibition of PACE4-altCT with C23 was able to inhibit growth of PCa cells both *in vivo* and *in vitro* [22]. Since C23 also resulted in decreased V-ATPase activity that was rescued with PRR overexpression, our current study suggests that PRR may be an important PACE4 substrate required for efficient tumor cell growth.

It is not yet clear why inhibition of PRR processing may lead to slower PCa growth. Inhibiting PACE4 leads to lower levels of both the secreted sPRR as well as the internal M8.9 fragment that remains associated with the V-ATPase (Fig 1). It is possible that sPRR may have pro-tumorigenic effects that act in an autocrine and/or paracrine manner that have yet to be discovered. Alternatively, reduced levels of the M8.9 fragment and subsequent lower V-ATPase activity would be predicted to have many negative effects on tumor cell growth. Intracellular acidification mediated by the V-ATPase is important for a wide array of processes that can enhance tumor growth including receptor mediated endocytosis, autophagy, mTOR signalling, and β-Catenin signalling (reviewed in [40]).

We have observed upregulation of PRR processing in both prostate [5] and breast cancer [23], however, another group has shown that sPRR levels are elevated in patients with pancreatic ductal adenocarcinoma (PDAC) and correlate with the clinical severity of the cancer [41]. Thus the role of PRR/sPRR may be a common feature of several cancer types and suggests an importance for PRR amount and processing in cell proliferation and tumorigenicity. Although multiple lines of evidence demonstrated that furin cleaves PRR [36,42], our findings point at PACE4 as a major protease of PRR, hinting at an important shift in the dynamic of PRR processing within PCa cells.

V-ATPase complex activity and loss of polarity in pancreatic ductal adenocarcinoma cells led to preferencial activation of different MMPs, favoring metastasis [43]. Plasma membrane activation of V-ATPase is linked to secreted cathepsin activation and to the metastatic potential of breast cancer cells [44]. Therefore, by cleaving PRR and increasing V-ATPase activity, PACE4 may not be replacing the function of other PRR proteases in PCa cells. Instead PACE4 may serve as an upstream activator of an oncogenic proteolytic cascade that increases the metastatic potential of PCa tumor cells.

## Conclusion

In this study, we expanded the knowledge on PRR cleavage and its contribution to the pathophysiology in PCa. Linking PTEN function with PACE4 and PRR cleavage in PCa cells warrants a better understanding of PC-substrate relationship during the development of therapeutics to target the appropriate proteases or substrates in cancer cells. We also hope that our work will warrant further investigation into the role of PACE4 in other malignancies, such as breast cancer and glioma, in which PRR role in tumorigeneis is documented.

## Supporting information

S1 Raw images (PDF)
