# Supplementary material for: Cleavage of the V-ATPase associated prorenin receptor is mediated by PACE4 and is essential for growth of prostate cancer cells
Source: PLoS One. 2023 Jul 18;18(7):e0288622. doi: 10.1371/journal.pone.0288622 (PMC10353799; doi:10.1371/journal.pone.0288622)

Figure 1 raw blots

Figure 1 A

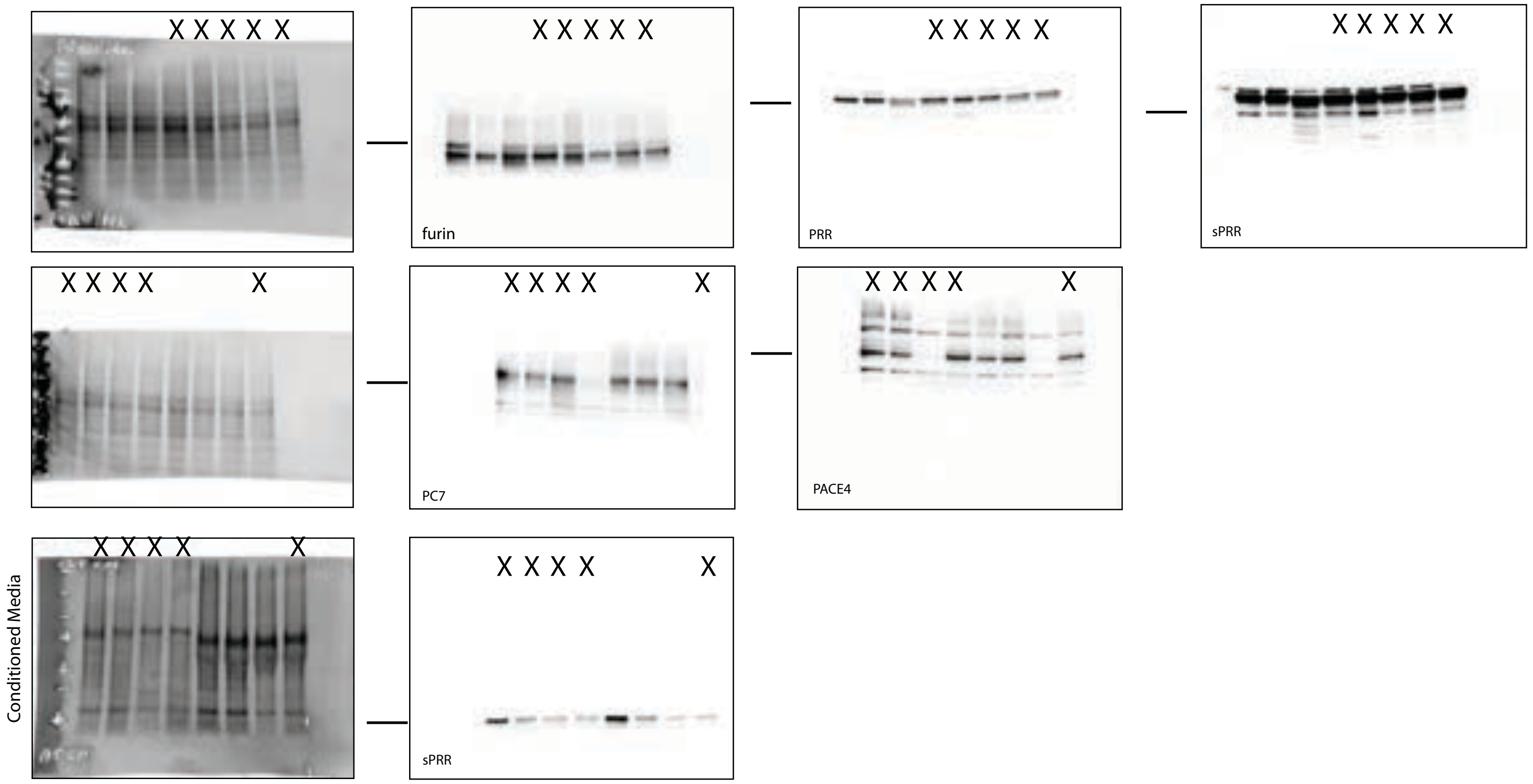

Figure 1 B

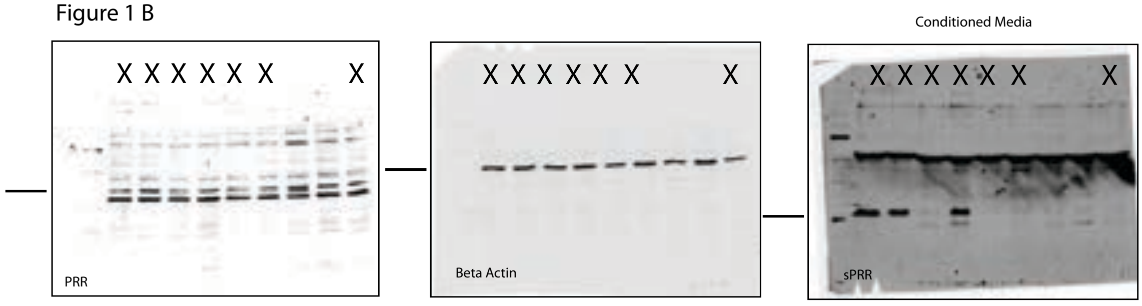

Figure 1 D

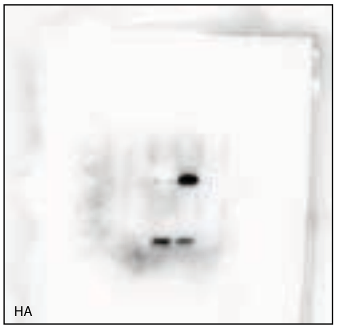

Figure 1 raw blots

Figure 1 G

Conditioned Media

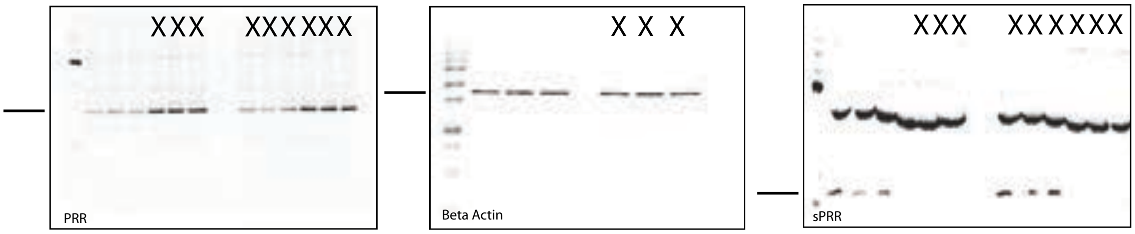

Figure 1 I

Conditioned Media

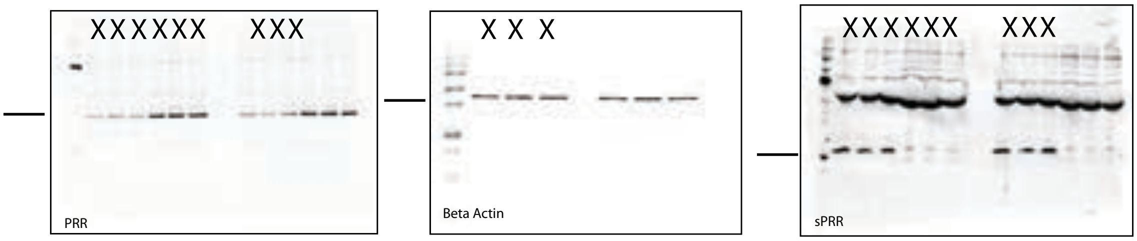

Figure 2 raw blots

Figure 2 A

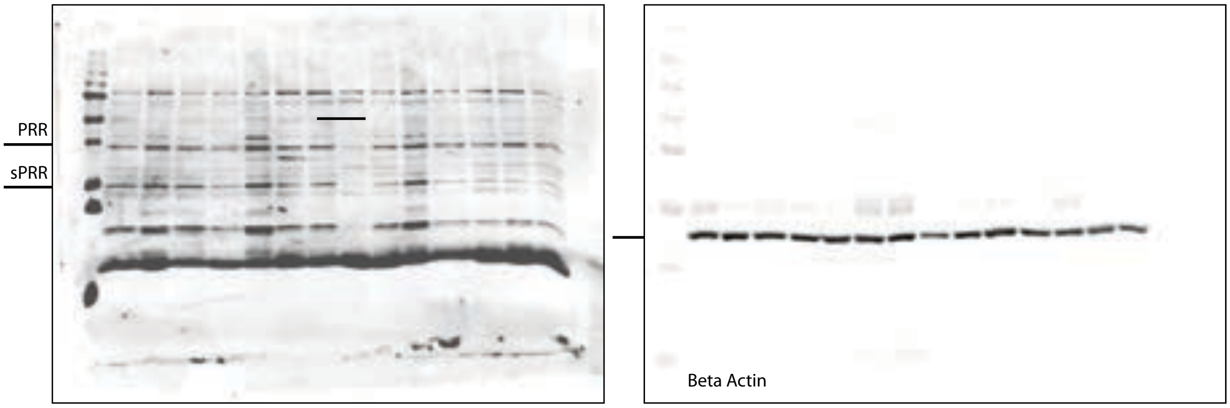

Figure 2 D

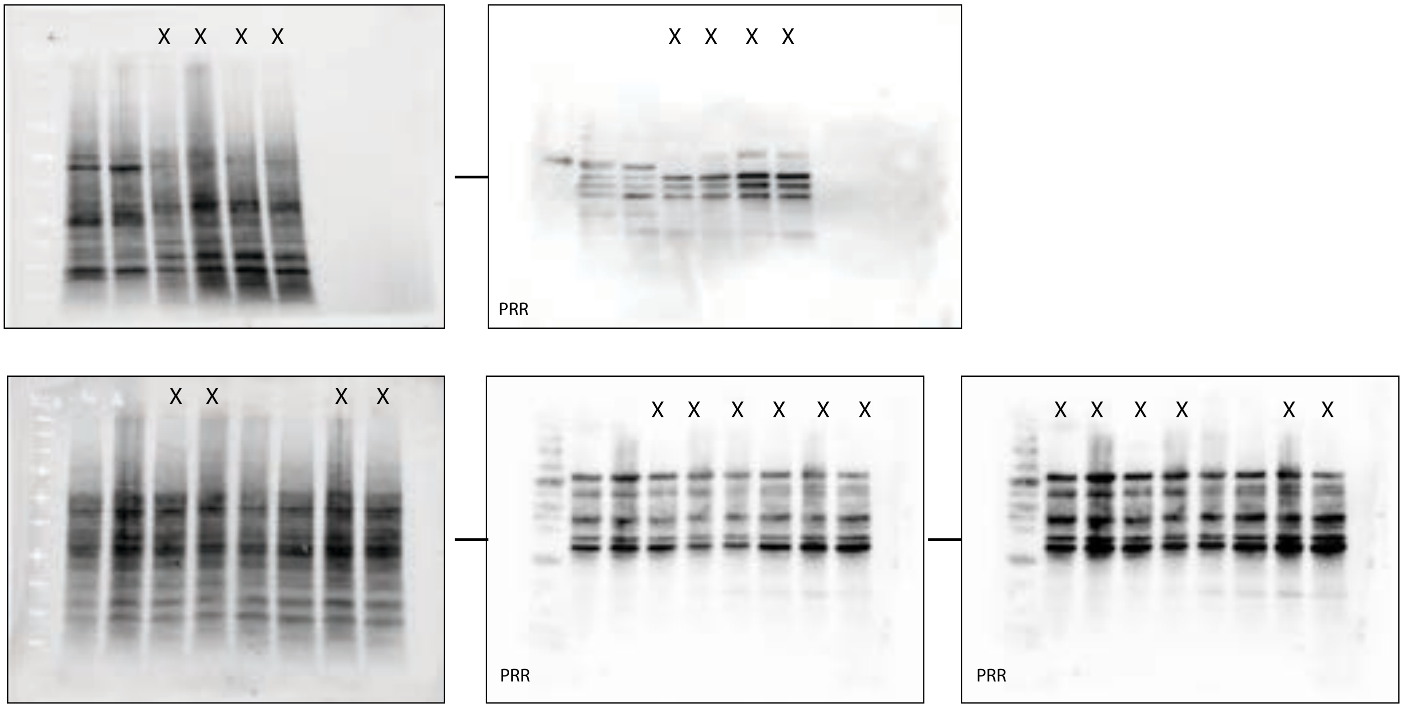

Figure 3 raw blots

Figure 3 A

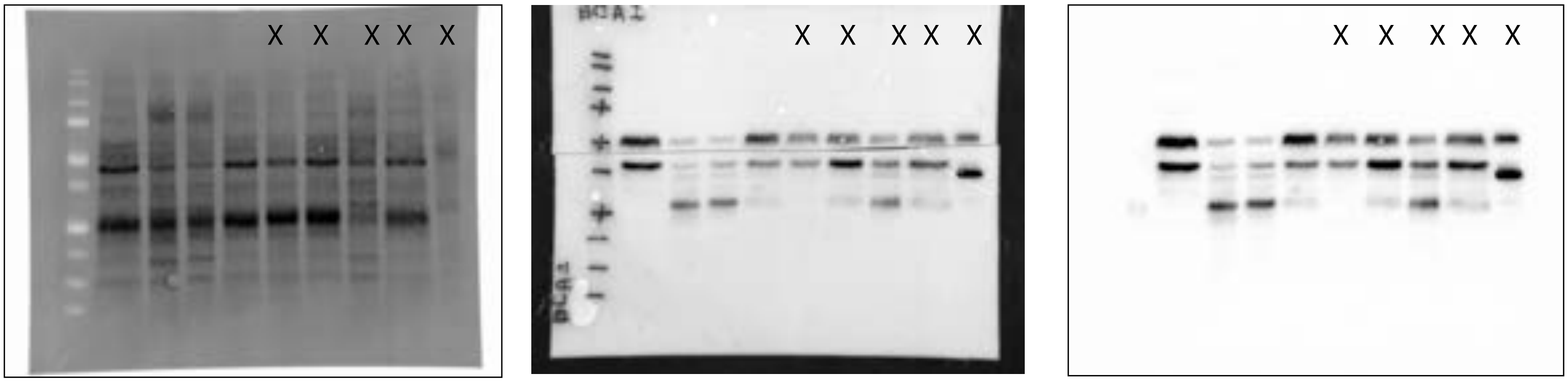

Figure 4 raw blots

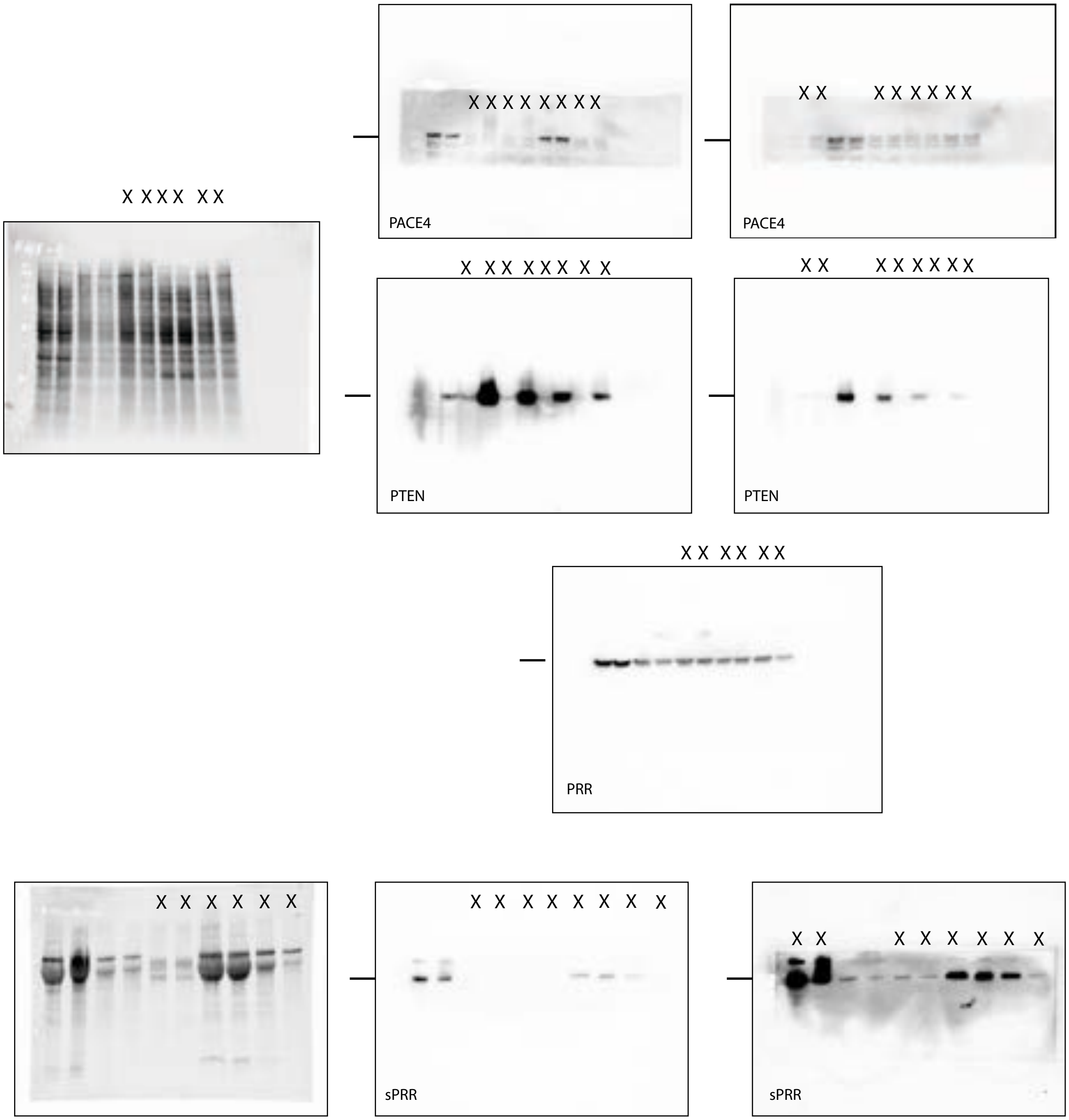

Figure 5 raw blots

Figure 5 B

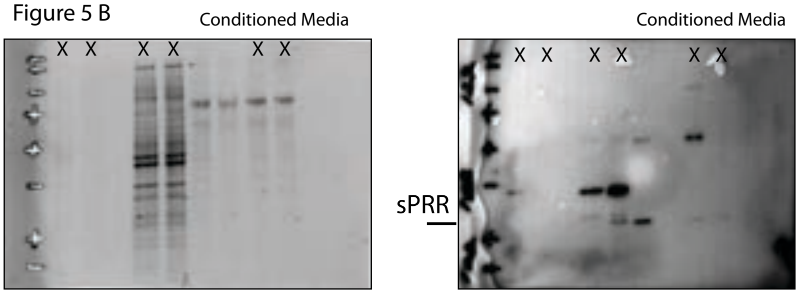

Figure 5 H

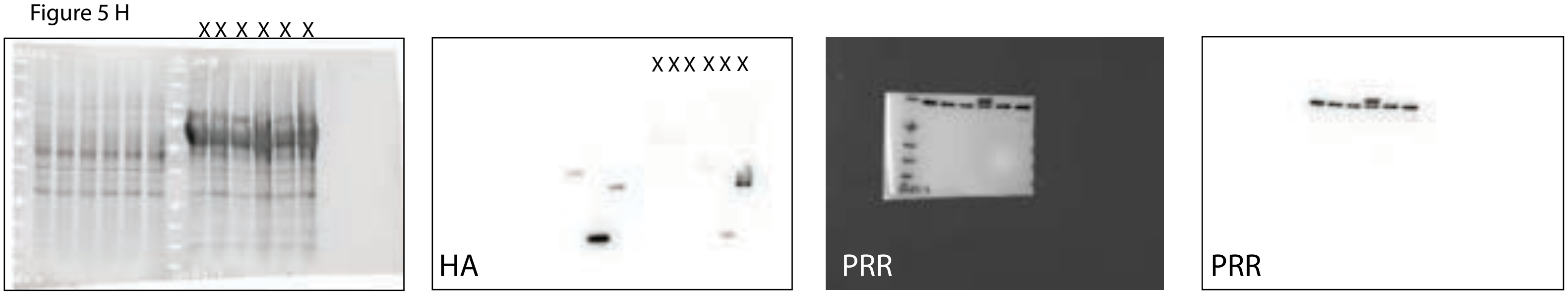

Supplement: S1 Raw images — (PDF) [file pone.0288622.s001.pdf]
